# [Corrigendum] MicroRNA‑92a contributes to tumor growth of human hepatocellular carcinoma by targeting FBXW7

**DOI:** 10.3892/or.2023.8596

**Published:** 2023-07-10

**Authors:** Wei Yang, Changwei Dou, Yufeng Wang, Yuli Jia, Chao Li, Xin Zheng, Kangsheng Tu

Oncol Rep 34: 2576–2584, 2015; DOI: 10.3892/or.2015.4210

Subsequently to the publication of the above paper, an interested reader drew to the authors' attention that certain of the control western blotting data featured in Fig. 5 on p. 2581 had also appeared in a couple of other articles featuring several of the same authors [Tu K, Dou C, Zheng X, Li C, Yang W, Yao Y and Liu Q: Fibulin-5 inhibits hepatocellular carcinoma cell migration and invasion by down-regulating matrix metalloproteinase-7 expression. BMC Cancer 14: 938, 2014; and Gai X, Tu K, Li C, Roberts LR and Zheng X: Histone acetyltransferase PCAF accelerates apoptosis by repressing a GLI1/BCL2/BAX axis in hepatocellular carcinoma. Cell Death Dis 6: e1712, 2015]. In addition, the authors drew to the attention of the Editorial Office that a couple of mistakes were made during the assembly of Fig. 2D on p. 2579.

The authors were able to re-examine their original data files, and realized that these figures had been inadvertently assembled incorrectly (they were also able to present the raw data from which these figures had been assembled to the Editorial Office). The revised versions of Figs. 2 and 5, containing the intended flow cytometric and western blotting data for these figures respectively, is shown on the next page. The authors wish to emphasize that the corrections made to these figures do not affect the overall conclusions reported in the paper, and they are grateful to the Editor of *Oncology Reports* for allowing them the opportunity to publish this corrigendum. All the authors agree to the publication of this corrigendum, and also apologize to the readership for any inconvenience caused.

## Figures and Tables

**Figure 2.**
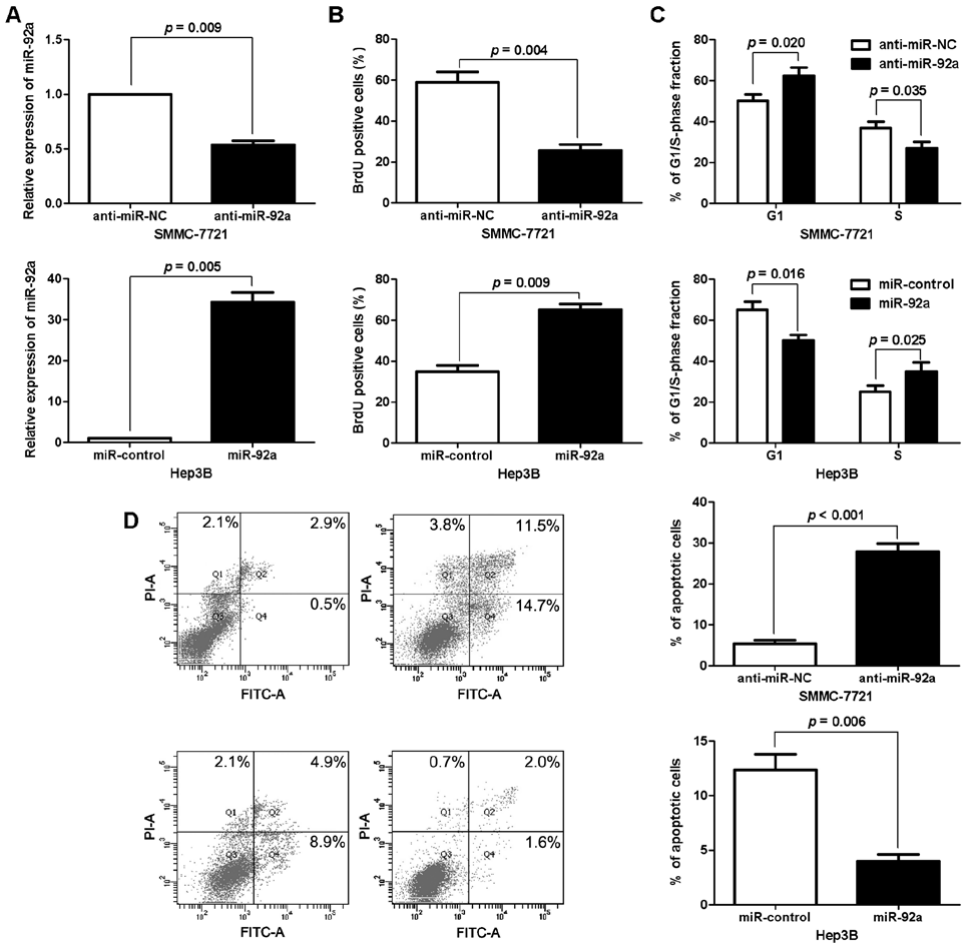
miR-92a promotes HCC cell proliferation, cell cycle transition and apoptosis resistance. (A) SMMC-7721 and Hep3B cells that were transfected with corresponding miRNA vectors were subjected to RT-qPCR for miR-92a. n = three independent experiments. (B) Cell proliferation as measured by BrdU incorporation assays was inhibited by knockdown of miR-92a in SMMC-7721 cells and increased by overexpression of miR-92a in Hep3B cells. n = three independent experiments. (C) As assessed by flow cytometry, knockdown of miR-92a induced G1 phase arrest in SMMC-7721 cells and the overexpression of miR-92a promoted cell cycle transition from G1 to S-phase in Hep3B cells. n = three independent experiments. (D) Quantification of the apoptotic cell population by flow cytometry. miR-92a knockdown increased the percentage of apoptotic SMMC-7721 cells and miR-92a-overexpressing Hep3B cells were composed of a smaller subset of apoptotic cells compared with the control cells. n = three independent experiments.

**Figure 5.**
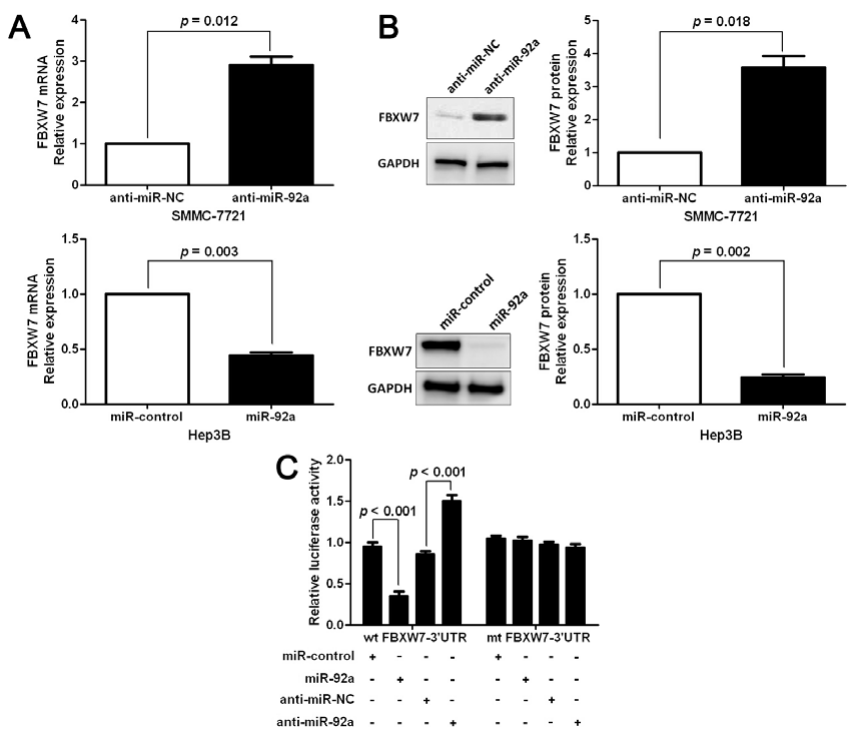
FBXW7 is identified as a direct target of miR-92a in HCC. (A) RT-qPCR analysis of FBXW7 mRNA expression in SMMC-7721 cells with anti-miR- 92a or anti-miR-NC vector transfection and Hep3B cells with miR-92a or miR-control vector transfection. n = three repeats with similar results. (B) Knockdown of miR-92a increases the level of FBXW7 protein in SMMC-7721 cells and overexpression of miR-92a reduced the expression of FBXW7 protein in Hep3B cells. n = three repeats with similar results. (C) miR-92a significantly suppresses the luciferase activity that carried wild-type (wt) but not mutant (mt) 3’-UTR of FBXW7. Anti-miR-92a led to a notable increase in the luciferase activity of wt 3’-UTR of FBXW7. n = three repeats with similar results.

